# Empowering Youth to Participate in Chemicals Management: A Youth Perspective

**DOI:** 10.1021/acs.est.6c03748

**Published:** 2026-05-26

**Authors:** Jonathan Blumenthal, Anna Shalin

**Affiliations:** † Department of Clinical and Biological Sciences, University of Turin, Turin 10124, Italy; ‡ Department of Earth Sciences, 7938 University of Toronto, Toronto, Ontario M5S 3B1, Canada

**Keywords:** chemical pollution, youth, science-policy interface, environmental justice, activism, planetary boundaries

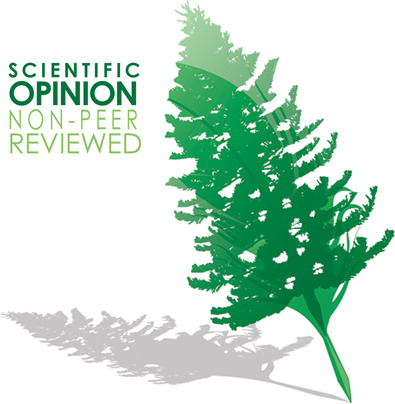

## Graphical abstract

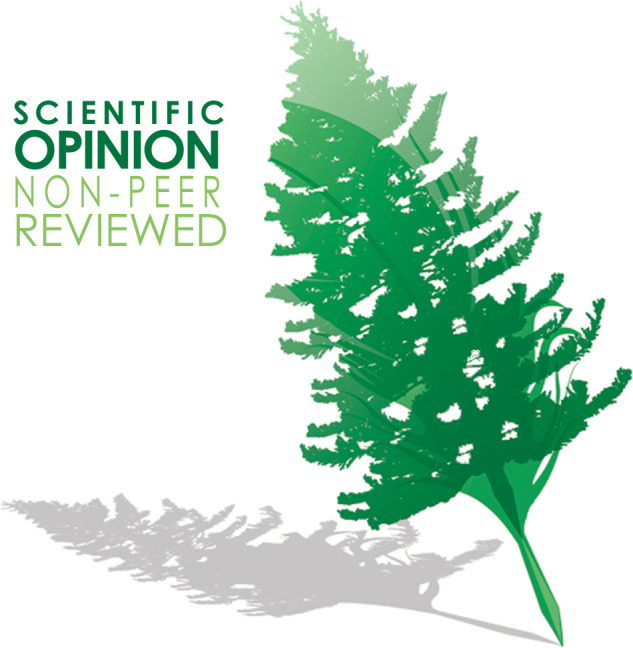

Chemical pollution, like climate change, is an issue with intergenerational impacts. The continued use and release of hazardous chemicals has been linked to adverse health effects, environmental degradation, and biodiversity loss – harms which disproportionately affect the health and well-being of today’s youth and of future generations.^1,2^ While we have witnessed large-scale youth activism on climate change, chemical pollution has not attracted the same attention. This Viewpoint, written by early career scientists, explores why chemical pollution has not inspired a comparable level of youth mobilization.

## The Unique Burden of Chemical Pollution on Youth

Today, virtually all humans are exposed to an unprecedentedly large volume and diversity of chemicals. Over 350,000 chemicals and mixtures are registered for production and use globally, of which only a small fraction have undergone rigorous safety evaluation.^3,4^ But from what few safety evaluations have been performed, there is evidence that exposure to certain chemicals is particularly detrimental during childhood and adolescence, with links to adverse health outcomes including obesity (e.g., bisphenol A, acrylamide, parabens),^5^ neurodevelopmental impairments (e.g., lead, certain pesticides),^6^ and asthma (e.g., polycyclic aromatic hydrocarbons, organophosphate insecticides).^7^ Incidences of certain cancers among children and young adults have also risen over the past few decades, even as they have declined among older adults, with exposure to chemicals considered as one possible risk factor.^8–10^ Chemical pollution may even have intergenerational health effects: for instance, epigenetic changes following parental exposure to certain pesticides and endocrine disruptors have been linked to the development of cancer in offspring.^11^

Chemical pollution is also altering our environment and surroundings on a larger scale, in a manner which risks depriving today’s youth, and future generations, of the right to live in a clean, healthy world. Chemical pollution contributes to a loss of biodiversity and ecosystem services which are difficult to reverse (e.g., loss of pollinators driven by neonicotinoid insecticides),^12,13^ while contamination of drinking water and soil (e.g., by per- and polyfluoroalkyl substances (PFAS)) requires expensive and resource-intensive remediation, if possible at all.^14,15^

## Elements Needed for Youth Mobilization

The impacts of chemical pollution on children and youth have been powerful driving forces in previous activist movements, even when not led by youth themselves. In 1978, for example, Lois Gibbs, concerned by her children’s health issues and believing them to be caused by pollution from the toxic chemicals buried under the local school, founded the Love Canal Homeowners Association (LCHA).^16^ The LCHA’s campaigning, in which many local children participated, contributed to the creation of the EPA’s Superfund program.^16^ In Grassy Narrows First Nation, which has suffered for over 50 years from mercury contamination, several generations of youth have organized events, protests, and media engagement to raise awareness of their community’s struggles.^17^

Yet, youth involvement in chemicals-related activism largely remains localized or tied to specific chemicals, and does not reflect a broader, large-scale movement addressing chemical pollution as a systemic, global problem.

To understand why chemical pollution as a systemic issue has not yet gained traction with youth activists, we look to the literature on the Fridays for Future movement, which mobilized youth around an environmental issue of similar scale. Here, we describe three elements present in the discussion surrounding climate change that were essential to the movement’s success but, as we demonstrate, are less established for chemical pollution (Figure 1).

**Figure 1:**
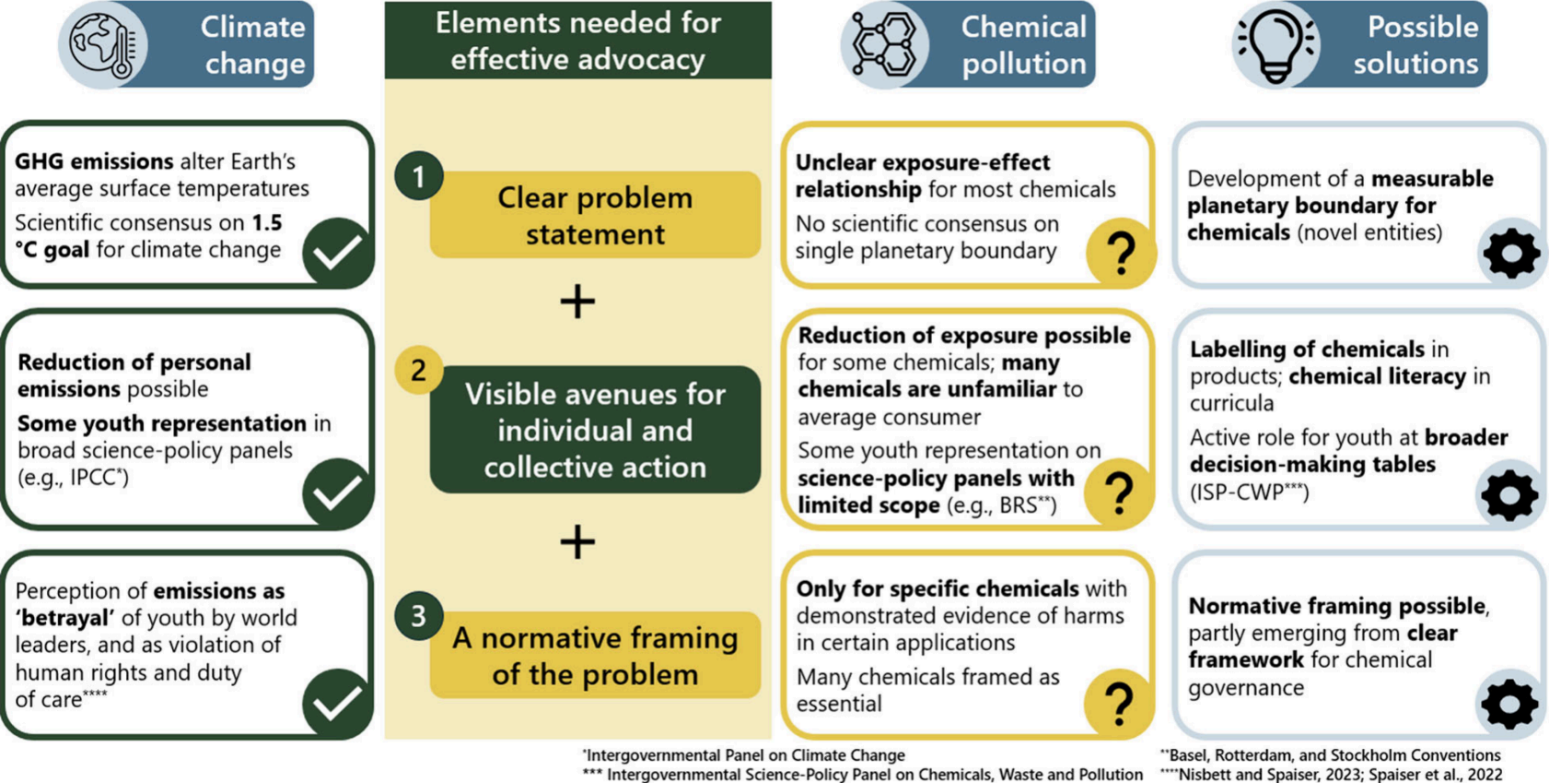
Overview of elements in the discussion around climate change that contributed to the success of the youth climate movement, their parallels for chemical pollution today, and possible solutions for encouraging and facilitating youth involvement based on this comparison.

### Element 1: Defining a Clear Problem Statement

One strength of the climate movement is the clarity of its core message: anthropogenic greenhouse gas emissions are driving global warming, which risks triggering irreversible planetary changes.

This message, grounded in a robust scientific consensus,^18^ can be communicated simply. The 1.5 °C limit for global warming described by the Intergovernmental Panel on Climate Change (IPCC) allows researchers, policymakers, activists, and other stakeholders to discuss the benefits and costs of specific actions (or inaction) using a common reference point.^19^

The relative complexity of chemical pollution (and lack of data on many chemicals)^3,20,21^ makes setting a similar target difficult, and thus complicates the ability to communicate impacts and possible remedies.^4^ Unlike greenhouse gases, which can be expressed in CO_2_ equivalents, no single expression of pressure or impact exists for chemicals. While some frameworks characterizing the burden of chemical exposures do exist – most notably disability-adjusted life years (DALYs) – their scope remains limited to a relatively small number of well-characterized chemicals, such as lead, and thus current DALY-based disease burden estimates are substantially underestimated (particularly in low- and middle-income countries, where exposure data are often sparse).^22^ Furthermore, the focus of DALYs on human health outcomes does not capture the broader ecological impacts of chemical pollution.

### Element 2: Creating Visible Avenues for Individual and Collective Action

While solutions to climate change are complex, the fundamental goal of reducing greenhouse gas emissions is widely understood. This clarity has also allowed young people to exercise eco-agency through actions like avoiding air travel and adopting plant-based diets, which contribute on a smaller scale to addressing the larger issue and allow individuals to feel empowered as agents of environmental and social change.^23^

For chemicals, clear solutions are harder to articulate. While it is possible to avoid exposure to certain chemicals in particular applications (e.g., avoiding nonstick PFAS cookware, or buying organic produce), there is limited transparency surrounding the chemical contents of products. The sheer number of chemicals, embedded across countless applications, also makes it difficult for individuals to understand which exposures they should prioritize avoiding, resulting in frustration and disengagement.

Similarly, multilateral chemical governance is fragmented across several conventions (e.g., Minamata, Stockholm), each addressing specific groups of chemicals. Although youth participation in these agreements has increased (e.g., through the UN Major Group for Children and Youth, or the Chemicals and Waste Youth Platform), the absence of a single coordinating body for chemical pollution comparable to the IPCC hinders meaningful engagement. With limited resources (funding, technical support, and time), youth face significant barriers to advocating across conventions negotiated in separate meetings worldwide, each with its own procedures.

### Element 3: Normative Framing

The Fridays for Future movement mobilized millions of youth in part due to its effective usage of normative framing.^24,25^ When appealing to their peers, the organizers of the Fridays for Future movement adapted the scientific consensus on climate change into a narrative of ‘right’ and ‘wrong.’^25^ Activists shared stories of childhoods overshadowed by the uncertainty of a planetary crisis, and their sense of betrayal by world leaders. In doing so, they grounded a complex scientific problem in human emotion and morality, taking advantage of well-established normative frames such as the duty of care for future generations.^24,25^ Similar appeals have driven some of the more successful movements against chemical pollution: much of Silent Spring’s power came from Rachel Carson’s clear link between the use of DDT and the death of baby birds.

However, these topics have been treated in a fragmented manner, and there is not a comparable approach to chemicals as a systemic issue. There is already a compelling narrative to be told: today’s youth are disproportionately exposed to harmful chemicals. We have inherited a system of chemical governance that has failed to protect us. As with climate change, the sense of betrayal can be a powerful catalyst for action. A normative framing for chemicals will also benefit from a clear planetary boundary, which provides a lens through which actions can be perceived as contributing to or hindering progress toward responsible chemicals management.

## Recommendations for Moving Forward

Youth activism has reshaped the discussion around climate change over the past decade and contributed to meaningful progress. The mounting evidence of harm from chemical pollution merits – and can achieve – a similar level of attention and engagement.

The **development of a numerical planetary boundary** (or series of boundaries) for novel entities is one way to achieve a clear problem statement. Like the 1.5 °C warming threshold for climate change, a planetary boundary for chemical pollution could be aspirational, helping distill complex science toward a tangible, if imperfect, rallying point.

The **integration of chemical pollution as a systems problem into educational curricula** is another avenue for introducing the issue in an accessible manner to youth: our generation grew up learning about climate change in school from various perspectives and therefore understands that it is not just a scientific problem but also a societal problem.

More **transparent, user-friendly labeling** of potentially hazardous chemicals in consumer products, paired with **teaching chemical literacy** in schools, would allow youth to identify chemicals in daily products and reduce personal exposure.

Finally, **youth must be empowered to cocreate visions for a chemical-safe future**. While technical solutions may not yet be fully defined, young people can contribute to shaping them. With respect to global decision-making processes, the recent establishment of the Intergovernmental Science-Policy Panel on Chemicals, Waste and Pollution (ISP-CWP, the equivalent of the IPCC for chemicals, waste, and pollution) presents one such opportunity. Allowing youth to have a dedicated seat on the Interdisciplinary Expert Committee (the Plenary’s scientific and technical advisory body), rather than solely participating as observers, would allow the panel’s recommendations to more fully reflect the needs and interests of the age demographic most affected by pollution.

On a national level, opportunities for youth engagement could be strengthened by integrating youth perspectives into domestic chemicals management frameworks (e.g., REACH in the EU, Canada’s Chemical Management Plan). This could take several forms, including the establishment of a youth council to advise legislators on the goals and requirements of the frameworks. Governments can also play a key role in addressing the financial and resource barriers which currently limit youth participation in multilateral processes, including through dedicated funding and mentorship, or through fellowship programs to support attendance and sustained engagement.

On a local level, one could envision eco-stewardship groups in schools increasing their focus on chemicals, and thus providing youth with opportunities to shape their local communities’ approach to the issue. Ultimately, we recognize that mechanisms for participation will vary by country and region, and we encourage further exploration of context-specific approaches to meaningful youth inclusion.

The youth-led climate movement has shown what is possible when a complex issue is made tangible, morally urgent, and action-oriented. Chemical pollution as a systemic problem, although more diffuse and less visible, carries similar stakes. We invite a broad, open, and constructive dialogue by researchers, policymakers, and interested youth on how best to empower and encourage youth to participate in the discussion.
